# Data on natural radionuclide's activity concentration of cement-based materials

**DOI:** 10.1016/j.dib.2020.106488

**Published:** 2020-11-05

**Authors:** M.M. Alonso, J.A. Suárez-Navarro, R. Pérez-Sanz, C. Gascó, A.M. Moreno de los Reyes, M. Lanzón, M.T. Blanco-Varela, F. Puertas

**Affiliations:** a Eduardo Torroja Institute for Construction Sciences, (IETcc-CSIC), Madrid-Spain; b CIEMAT, Madrid-Spain; c Departamento de Arquitectura y Tecnología de la Edificación, ETSAE, Universidad Politécnica de Cartagena (UPCT), Cartagena, Spain; d TYPSA (Técnica y Proyectos S.A.)

**Keywords:** Radioactivity, Cement based materials, Supplementary cementitious materials, Chemical composition, NORM wastes

## Abstract

Cement based materials may contain varying levels of radionuclides, mainly ^226^Ra (from the ^238^U series), ^232^Th and ^40^K, which are used to determine the Activity Concentration Index ("ACI"). According to the European directive Euratom 2013/59 in these materials, the “ACI” must be < 1 to be suitable for their use in construction. In this paper, data on the activity concentration of natural radionuclides in cement-based materials (i.e. cements, additions, pigments and aggregates) as well as their chemical composition are presented. Radioactivity measurements have been determined by using gamma spectroscopy the chemical compositions have been determined by X-Ray Fluorescence. Data for cements measured shown that white cements present a lower concentration of activity than conventional CEM I. In addition, the CAC (Calcium aluminate cements) present high activity concentration in the ^232^Th series. Regarding additions, FA (Fly Ash) are those that present the highest concentration of activity in the ^238^U and ^232^Th series, while olive biomass ashes are those supplementary cementitious materials that show the highest concentration of activity for ^40^K. Some pigments used in mortar and concrete technology were also characterized. Granitic and volcanic rocks, potentially used as aggregates present much higher activity concentration than the siliceous aggregate.

## Specifications Table

**Table:** 

Subject	Physics
Specific subject area	Radioactivity and Radiological hazards in cement based materials
Type of data	Tables
How data were acquired	The chemical oxide compositions of the materials were determined on S8 Tiger Bruker X-ray fluorescence (XRF) spectrometer. Loss on ignition was also calculated as per European standard EN196-2:2014 [1] Radiological measurements of the samples were carried out by means of gamma ray spectrometry using a system with two high purity germanium detectors one of them is coaxial (Type p), and the other are BEGe (Broad Energy Germanium detector).
Data format	Raw
Parameters for data collection	For FRX measurements, the samples were dried and subsequently ground to ensure a particle size under 63 microns. For radiological measurements of the samples, and in order to ensure secular equilibrium between ^226^Ra and ^232^Th and their progenies, a plastic cylindrical beaker containers with 75.4 mm of diameter and 31 mm height were used. They were completely filled with each sample, hermetically sealed to avoid ^222^Rn losses and let stand for at least 25 days before their measurement. In all cases samples were measured by duplicate.
Description of data collection	The determination of chemical composition of materials by XRF was carried out in pellets or fuse beads. The radionuclides determined in this study were those belonging to the natural radioactive series of uranium, actinium and thorium along with ^40^K. The gamma emitters determined in the uranium series were: ^234^Th (63 keV), ^214^Pb (351 keV), ^214^Bi (609, 1120 and 1765 keV), ^226^Ra (186 keV eliminating interference from ^235^U), ^210^Pb (46.5 keV). In the actinium series the activity of ^235^U (144 and 163 keV) was determined. For the thorium series the gamma emitters analyzed were: ^228^Ac (911 keV), ^212^Pb (238 keV) and ^208^Tl (583 keV). The activity concentration of ^40^K was determined by its characteristic photopeak at 1460 keV. The efficiency calibration of the gamma-ray detectors was computed by using the mathematical code LabSOCS (InSitu Object Calibration Software). The samples were measured for 80,000 s to minimize the measurement uncertainty and achieve the required detection limits. The spectra were analysed using an Excel spreadsheet and Genie 2000 software. Samples were measured in duplicate, except in cases where not enough sample was available.
Data source location	The samples are from different sources, mainly from Spain and other European countries. The samples were collected by both the IETcc-CSIC and the UPCT. All the samples were registered and centralized in the IETcc-CSIC where they were chemically characterized. An aliquot of the samples was sent to CIEMAT for the determination of the gamma emitters by means of high resolution gamma spectrometry (HPGe).
Data accessibility	With the article

## Value of the Data

• The data presented herein can be used as an on-line database of the natural radioactivity of cement-based materials
• The data can be used for radiological studies, as well as for dose rate calculations and the Activity Concentration Index of each of the cement based materials.
• The results can be the basis for the calculation of the activity concentration or excess effective dose rates described in the European Union Directive 2013/59 [2] for final cement based materials.

## 1 Data Description

### 1.1 Chemical composition of cement based materials

This section presents the chemical composition of all materials along with their loss on ignition. A brief description of the materials accompanies these data.

#### 1.1.1 Cements

Chemical composition of 16 different cements (Portland and other types of cements) is shown in Table 1.

**Table 1:** Chemical composition (wt %) of selected cements.

Sample	Cements	SiO_2_	CaO	Al_2_O_3_	Fe_2_O_3_	MgO	MnO	Na_2_O	K_2_O	TiO_2_	SO_3_	Others	LoI	Total
C1	CEM I 42,5R	18.72	62.98	5.63	2.68	0.87	0.05	0.04	0.85	0.23	3.05	0.16	2.31	97.57
C2	CEM I 52,5R	20.50	57.00	5.40	2.10	3.70	0.02	0.60	1.40	0.20	6.40	0.10	2.30	99.72
C3	CEM I 52,5R	19.65	63.52	5.24	2.78	0.85	0.06	0.12	1.04	0.23	3.40	0.06	2.90	99.85
C4	CEM I 52,5R	20.88	61.41	4.96	2.65	1.80	0.02	0.69	1.18	0.17	3.11	0.13	2.94	99.94
C5	CEM I 52,5R	19.36	62.32	4.42	2.62	1.86	-	0.09	0.94	0.23	3.23	0.4	2.75	98.22
C6	CEM I 52,5R	20.29	64.47	5.67	2.35	0.84	0.06	0.11	0.97	0.24	2.91	0.25	2.97	101.13
C7	CEM II/A-L 42.5R	18.36	62.16	4.15	3.30	2.02	-	0.03	0.24	0.38	3.04	0.05	5.40	99.13
C8	CEM II/B-V 42.5N	28.26	49.28	8.95	4.32	1.90	0.06	0.37	0.91	0.56	2.64	0.43	1.86	99.54
C9	CEM III/B 42.5N	30.67	46.21	9.10	1.17	5.55	0.13	0.20	0.70	0.80	4.93	0.05	-	99.51
C10	CEM I 52,5 S/R	19.83	62.70	4.02	4.28	0.73	0.02	0.07	0.63	0.18	2.72	0.16	3.24	98.58
C11	White Cement	19.00	68.00	3.30	0.30	1.80	1.70	1.30	0.40	-	3.60	0.10	1.80	101.3
C12	White Cement	20.10	65.84	4.11	0.27	0.40	-	-	0.73	-	3.53	0.14	3.30	98.42
C13	CAC	3.30	33.50	44.90	15.00	0.80	-	0.30	-	1.50	-	-	0.20	99.50
C14	CAC	4.15	38.77	37.92	15.56	0.68	-	-	0.12	1.96	-	0.25	0.08	99.49
C15	CAC	4.24	38.07	37.81	14.74	0.66	-	-	0.11	1.99	-	0.37	0.06	98.05
C16	Calcium sulfoaluminate	8.14	41.50	23.20	1.05	3.22	0.10	0.86	0.44	0.32	18.36	1.37	1.45	100.01

LoI: Loss on Ignition

#### 1.1.2 Supplementary Cementitious Materials (SCMs)

Table 2 presents the chemical composition data of three different types of SCMs a): FA (Fly Ash); b): S (Slags- where S1 to S3 are blast furnace slags and S4-S5 are steel slags) and c): other SCMs (SF- Silica Fume, G-Waste Glass, L-Limestone, MK-Metakaolin, PZ- Pozzolans, OBBAM- Olive Biomass Bottom Ashes Mixed and OBFAM- Olive Biomass Fly Ashes Mixed

**Table 2:** Chemical composition (wt %) of different Supplementary Cementitious Materials.

SCM	SiO_2_	CaO	Al_2_O_3_	Fe_2_O_3_	MgO	MnO	Na_2_O	K_2_O	TiO_2_	SO_3_	Others	LoI	Total
FA1	46.30	4.90	31.00	4.50	1.30	0.10	0.30	1.30	1.50	1.00	1.00	6.80	100
FA2	44.65	3.88	24.50	6.85	1.88	0.09	0.75	3.40	3.40	1.73	0.49	10.72	102.34
FA3	49.40	3.49	23.48	7.20	1.64	-	0.75	4.25	1.03	-	1.35	6.15	98.74
FA4	35.61	14.52	14.84	12.56	4.03	-	1.14	1.36	0.95	-	0.96	12.30	98.27
FA5	39.03	6.40	27.06	19.50	1.04	-	0.16	1.41	0.96	-	0.85	1.82	98.23
FA6	48.85	5.52	28.66	4.52	1.01	-	0.54	1.31	1.59	-	-	4.14	96.14
FA7	54.90	3.41	20.80	6.75	1.90	-	1.29	2.33	0.97	-	0.28	5.85	98.48
FA8	54.44	2.72	27.51	6.38	1.51	-	1.51	3.13	-	-	0.70	2.10	100
FA9	47.97	4.30	31.03	5.07	1.92	0.02	1.28	1.32	1.20	0.14	0.45	5.30	100
FA10	42.44	4.78	26.95	18.40	0.80	0.05	0.50	1.53	1.07	1.44	0.43	1.63	100.02
FA11	54.60	4.21	25.46	5.05	1.21	0.06	0.46	1.35	1.47	0.61	1.20	4.30	99.98
S1	35.30	41.00	13.60	0.40	4.10	-	0.01	-	-	-	-	2.70	97.11
S2	39.21	40.28	10.36	0.33	7.65	0.21	0.67	0.35	0.36	1.04	-	-	100.46
S3	34.83	37.86	11.58	0.19	11.90	0.26	0.01	0.28	0.53	1.91	0.35	0.30	100.01
S4	29.98	45.84	6.04	0.92	10.60	1.49	-	-	1.43	0.31	2.62	0.81	100.04
S5	29.07	47.37	6.20	0.87	9.28	1.28	-	-	1.37	0.40	2.34	1.83	100.01
SF1	94.30	0.50	0.20	0.10	0.20	0.02	0.10	0.40	-	0.10	0.05	4.00	99.97
G1	70.70	11.80	2.00	0.50	1.20	-	11.70	1.10	0.10	-	0.04-	-	99.14
L1	0.30	54.60	0.04	0.10	0.90	0.01	0.40	-	0.01	-	0.10	43.60	100.06
RM1	9.77	10.40	14.56	36.96	0.17	0.03	5.58	0.12	5.24	0.30	1.07	15.80	100
MK1	52.80	0.16	32.40	0.49	0.09	-	-	0.89	0.17	0.02	0.14	12.90	100.06
MK2	54.24	0.12	44.06	0.38	0.12	-	-	0.68	0.15	-	0.05	0.17	99.97
PZ1	45.05	11.07	13.46	13.20	6.83	0.17	1.99	1.15	3.30	0.04	1.08	2.65	99.99
PZ2	44.50	10.72	13.97	13.40	6.30	0.16	1.48	1.78	3.29	0.06	1.22	3.11	99.99
PZ3	38.72	15.36	11.61	13.27	7.59	0.17	0.60	0.72	3.23	0.06	1.54	7.13	100
OBBAM1	23.61	20.61	5.41	6.12	6.02	0.10	1.29	17.31	0.83	0.80	5.30	13.4	99.99
OBFAM1	12.61	20.21	2.97	2.3	4.85	0.06	1.12	26.34	0.24	4.00	6.90	18.4	100.00

LoI: Loss on Ignition

#### 1.1.3 Pigments used in mortar and concrete technology

Chemical composition of seven mineral pigments used in building materials is shown in Table 3.

**Table 3:** Chemical composition (wt %) of different mineral pigments.

Pigments	Color	SiO_2_	CaO	Al_2_O_3_	Fe_2_O_3_	Fe_3_O_4_	MgO	MnO	Na_2_O	K_2_O	TiO_2_	SO_3_	Cr_2_O_3_	Other oxides	FeOOH	S^−^	Total
P1	Blue	38.00	0.33	25.00	1.20	-	0.11	-	22.00	2.77	0.20	-	-	0.27	-	13.00	102.88
P2	Green	0.07	0.15	-	0.04	-	-	-	0.14	-	-	0.39	98.94	0.09	-	-	99.82
P3	Black	0.31	-	0.11	-	94.70	-	2.04	-	-	1.42	0.56	-	0.56	-	-	99.70
P4	Red	0.14	-	0.08	98.86	-	-	0.01	-	-	-	0.62	-	0.57	-	-	100.28
P5	Red	5.94	4.15	3.29	82.62	-	2.38	0.07	-	0.74	0.10	0.02	-	0.09	-	-	99.4
P6	Yellow	0.05	-	0.07	-	-	-	0.01	-	-	-	0.79	-	0.14	98.78	-	99.84
P7	White	0.08	-	3.94	0.01	-	-	-	-	-	95.51	0.03	-	0.27	-	-	99.85

LoI: Loss on Ignition

#### 1.1.4 Aggregates used in mortar and concretes preparation

Finally, six aggregates with particle size between 0–2 mm (one siliceous- A1, three granitic ones A2, A3, A4 and two volcanic ones A5 and A6) used in the preparation of mortars and concretes have been chemically characterised. Results are shown in Table 4.

**Table 4:** Chemical composition (wt %) of six aggregates (size 0-2 mm).

Aggregates	SiO_2_	CaO	Al_2_O_3_	Fe_2_O_3_	MgO	MnO	Na_2_O	K_2_O	TiO_2_	P_2_O_5_	Others	LoI	Total
A1	96.80	0.10	1.50	0.40	-	-	-	0.60	-	-	-	0.60	100
A2	73.51	0.86	14.12	1.65	0.39	0.05	3.15	4.31	-	-	-	0.5	99.46
A3	74.6	1.3	12.77	2.5	2.84	0.06	2.71	4.7	-	-	-	2.9	99.15
A4	69.72	2.84	12.77	3.08	0.63	0.04	2.56	7.19	-	-	-	1.1	98.84
A5	45.14	8.60	13.20	10.87	7.33	0.22	4.33	2.33	2.94	0.84	0.60	3.65	100.08
A6	45.86	9.86	13.10	9.41	5.80	0.18	4.26	2.43	2.55	0.72	0.61	5.20	99.98

LoI: Loss on Ignition

### 1.2 Activity concentrations of series ^238^U, ^235^U, ^232^Th and ^40^K in cement based materials

The activity concentrations of ^238^U, ^235^U and ^232^Th series as well as and ^40^K given in Bq kg^−1^ in all measured cement based materials are shown below. The uncertainties of the activity concentrations shown in Tables 5, 6, 7 and 8 were calculated from the measurement uncertainties (peak area and counting efficiency). The uncertainty associated with the weight of the samples was considered negligible.

**Table 5:** Activity concentration for the main gamma emitters in the naturally occurring ^238^U, ^235^U, ^232^Th and ^40^K series in cements.

Series		^238^U Radiactive Serie					^235^U	^232^Th Radiactive Serie			^40^K
Cements		^234^Th	^226^Ra	^214^Pb	^214^Bi	^210^Pb		^228^Ac	^212^Pb	^208^Tl	
C1	a	16.9 ± 4.2	18.8 ± 4.5	19.4 ± 3.0	15.0 ± 1.1	< 11.2	-	19.0 ± 1.4	21.1 ± 3.4	6.21 ± 0.79	185 ± 16
	b	19.8 ± 4.6	16.8 ± 4.3	18.4 ± 2.9	14.8 ± 1.3	12.9 ± 5.7	-	17.9 ± 1.6	20.6 ± 3.4	6.33 ± 0.84	183 ± 17
C2	a	19.6 ± 1.9	-	19.3 ± 0.8	-	-	-	20.1 ± 1.0	19.16 ± 0.3	5.5 ± 0.4	237.5 ± 7.1
	b	20.0 ± 2.2	-	19.2 ± 0.8	-	-	-	17.8 ± 1.1	19.1 ± 0.6	6.2 ± 0.4	238.3 ± 7.5
C3	a	-	-	15.8 ± 1.0	14.94 ± 0.80	-	-	18.6 ± 1.6	17.06 ± 0.96	5.47 ± 0.47	262 ± 130
C4	a	21.8 ± 3.1	18.7 ± 5.5	20.6 ± 3.4	19.1 ± 1.4	17.6 ± 4.4	-	19.0 ± 1.8	21.0 ± 3.4	7.52 ± 0.73	201 ± 19
	b	20.1 ± 3.2	21.9 ± 5.8	21.5 ± 3.6	19.7 ± 1.3	19.3 ± 3.2	-	20.7 ± 1.7	23.7 ± 3.9	8.47 ± 0.77	223 ± 20
C5	a	30.3 ± 6.3	30.7 ± 5.6	32.6 ± 4.9	26.4 ± 1.5	30.1 ± 8.8	1.18 ± 0.37	16.9 ± 1.1	< 0.5	5.45 ± 0.67	195 ± 17
	b	31.6 ± 6.6	31.6 ± 5.8	31.8 ± 4.8	26.5 ± 1.6	29.9 ± 9.5	1.39 ± 0.51	16.5 ± 1.1	15.3 ± 2.6	5.52 ± 0.68	204 ± 18
C6	a	22.1 ± 3.3	16.8 ± 5.4	18.1 ± 3.0	16.8 ± 1.2	19.3 ± 4.6	-	20.0 ± 1.9	18.8 ± 3.1	7.89 ± 0.76	234 ± 21
	b	18.3 ± 3.2	21.1 ± 5.5	19.4 ± 3.2	17.8 ± 1.1	18.9 ± 3.4	-	20.3 ± 1.8	23.4 ± 3.8	8.63 ± 0.77	236 ± 21
C7	a	26.9 ± 5.7	26.1 ± 5.0	27.5 ± 4.2	21.8 ± 1.3	27.5 ± 8.1	-	5.99 ± 0.46	5.9 ± 1.1	1.88 ± 0.24	44.0 ± 4.0
	b	27.1 ± 5.7	27.4 ± 5.2	26.7 ± 4.1	21.9 ± 1.4	27.3 ± 8.9	-	6.02 ± 0.55	5.7 ± 1.0	1.95 ± 0.27	43.4 ± 4.3
C8	a	97 ± 20	84 ± 23	91 ± 14	87.5 ± 5.3	< 10.6	-	51.1 ± 3.7	52.2 ± 8.4	20.3 ± 2.6	190 ± 18
	b	103 ± 15	79 ± 19	95.3 ± 5.3	90.2 ± 3.2	< 5.6	3.8 ± 1.1	49.8 ± 2.3	53.6 ± 3.7	20.8 ± 1.1	173 ± 10
C9	a	79 ± 18	68 ± 19	77 ± 12	72.0 ± 4.5	62 ± 19	3.3 ± 1.2	33.6 ± 2.8	36.0 ± 5.9	13.5 ± 1.7	211 ± 19
	b	44.0 ± 4.7	64.5 ± 7.8	58.0 ± 2.0	61.4 ± 1.9	24.4 ± 3.4	-	29.1 ± 1.7	26.6 ± 1.1	11.03 ± 0.65	213 ± 10
C10	a	16.3 ± 4.1	20.3 ± 4.7	17.1 ± 2.6	13.7 ± 1.0	18.8 ± 6.0	-	14.0 ± 1.1	15.9 ± 2.6	4.71 ± 0.61	147 ± 13
	b	18.4 ± 4.3	± 18.8 ± 4.4	17.4 ± 2.7	13.8 ± 1.2	16.0 ± 6.4	-	12.9 ± 1.2	15.6 ± 2.5	4.62 ± 0.62	140 ± 13
C11	a	54.2 ± 3.2	-	56.0 ± 1.2	-	-	-	4.77 ± 0.7	-	4.05 ± 0.5	1.27 ± 0.1
	b	62.8 ± 5.5	-	58.3 ± 1.1	-	-	-	4.85 ± 0.8	-	4.68 ± 0.3	1.45 ± 0.2
C12	a	23.5 ± 5.4	23.9 ± 5.4	23.7 ± 3.6	18.7 ± 1.3	25.1 ± 7.8	-	16.5 ± 1.3	17.7 ± 2.9	5.33 ± 0.68	144 ± 13
	b	23.9 ± 5.3	23.1 ± 4.8	23.2 ± 3.5	18.5 ± 1.2	25.9 ± 7.7	-	15.8 ± 1.1	17.9 ± 3.0	5.11 ± 0.64	149 ± 13
C13	a	75 ± 8.5	-	65.4 ± 1.4	-	-	-	130.4 ± 2.1	-	136.4 ± 1.8	41.7 ± 0.8
	b	72.5 ± 9.0	-	64.4 ± 1.3	-	-	-	132.2 ± 2.3	-	138.4 ± 1.5	43.2 ± 0.8
C14	a	82 ± 17	85 ± 16	83 ± 13	65.9 ± 3.8	24 ± 20	4.9 ± 2.0	113.6 ± 7.5	123 ± 20	36.5 ± 4.5	-
	b	81 ± 17	82 ± 15	82 ± 12	66.9 ± 3.7	41 ± 12	3.95 ± 0.76	112.9 ± 7.4	122 ± 20	36.5 ± 4.4	-
C15	a	87 ± 18	80 ± 15	82 ± 12	68.0 ± 4.0	44 ± 14	4.5 ± 1.2	109.9 ± 7.3	118 ± 19	35.6 ± 4.4	-
	b	76 ± 16	87 ± 16	83 ± 13	66.1 ± 3.9	44 ± 13	-	110.5 ± 7.4	118 ± 19	33.9 ± 4.2	-
C16	a	9.8 ± 2.8	17.3 ± 4.1	15.2 ± 2.4	11.9 ± 1.0	13.2 ± 4.4	-	4.74 ± 0.66	5.32 ± 0.90	1.49 ± 0.23	105 ± 10
	b	15.6 ± 3.8	14.0 ± 3.9	14.5 ± 2.3	11.4 ± 1.1	13.1 ± 5.8	-	4.84 ± 0.76	4.99 ± 0.86	1.54 ± 0.28	105 ± 11

Uncertainties are quoted for a coverage factor k = 2 and are due to measurement uncertainties.

**Table 6:** Activity concentration for the main gamma emitters in the naturally occurring ^238^U, ^235^U, ^232^Th and ^40^K series in different mineral additions.

Series		^238^U Radiactive Serie					^235^U	^232^Th Radiactive Serie			^40^K
SCM		^234^Th	^226^Ra	^214^Pb	^214^Bi	^210^Pb		^228^Ac	^212^Pb	^208^Tl	
FA1	a	131 ± 10	-	128.5 ± 1.8	-	-	-	129.6 ± 1.9		133.4 ± 2.0	40.0 ± 0.7
	b	129 ± 10	-	126.2 ± 1.9	-	-	-	131.0 ± 2.4		134.2 ± 1.6	42.7 ± 0.9
FA2	a	87.5 ± 9.0	88.4 ± 3.4	88.4 ± 3.4	79.7 ± 2.3	-	-	85.1 ± 3.0	76.6 ± 3.0	24.0 ± 0.9	868 ± 32
FA3	a	90 ± 19	91 ± 16	91 ± 14	75.6 ± 4.5	85 ± 25	-	81.7 ± 5.5	88 ± 14	26.8 ± 3.3	853 ± 73
	b	86 ± 18	92 ± 17	91 ± 14	74.7 ± 4.3	88 ± 26	-	83.0 ± 5.5	89 ± 15	26.5 ± 3.2	865 ± 74
FA4	a	46 ± 10	59 ± 11	57.7 ± 8.8	46.0 ± 2.8	50 ± 15	-	44.2 ± 3.1	47.3 ± 7.9	14.5 ± 1.8	214 ± 19
	b	56 ± 12	49 ± 10	57.6 ± 8.8	45.6 ± 3.0	51 ± 15	-	41.9 ± 3.1	46.9 ± 7.7	14.5 ± 1.8	215 ± 20
FA5	a	137 ± 28	129 ± 24	130 ± 20	105.7 ± 6.3	128 ± 38	7.5 ± 1.9	62.2 ± 4.5	67 ± 11	21.6 ± 2.7	342 ± 31
	b	131 ± 27	133 ± 24	129 ± 20	105.5 ± 6.0	125 ± 37	6.1 ± 1.6	63.1 ± 4.3	68 ± 11	20.5 ± 2.5	346 ± 30
FA6	a	148 ± 31	148 ± 27	144 ± 22	118.9 ± 7.3	128 ± 37	-	142 ± 10	150 ± 25	46.0 ± 5.7	232 ± 22
	b	138 ± 29	151 ± 28	147 ± 22	118.0 ± 6.8	128 ± 38	-	138.5 ± 9.3	148 ± 24	44.3 ± 5.4	230 ± 21
FA7	a	69 ± 15	75 ± 14	72 ± 11	59.3 ± 3.6	74 ± 22	-	47.3 ± 3.3	51.6 ± 8.6	15.9 ± 2.0	482 ± 42
	b	81 ± 17	64 ± 13	72 ± 11	59.4 ± 3.8	70 ± 21	-	47.7 ± 3.5	51.4 ± 8.5	15.6 ± 2.0	482 ± 43
FA8	a	63 ± 13	65 ± 12	68 ± 10	55.2 ± 3.2	58 ± 17	-	87.8 ± 5.8	94 ± 15	28.0 ± 3.4	659 ± 56
	b	66 ± 14	69 ± 13	69 ± 11	55.0 ± 3.4	56 ± 17	-	87.5 ± 5.9	88 ± 14	28.9 ± 3.6	643 ± 56
FA9	a	84 ± 18	74 ± 14	80 ± 12	64.4 ± 3.8	70 ± 21	-	49.6 ± 3.4	53.1 ± 8.8	15.5 ± 1.9	269 ± 24
	b	89 ± 19	76 ± 15	70.1 ± 9.2	64.7 ± 4.1	75 ± 22	4.7 ± 1.9	49.0 ± 3.6	49.9 ± 8.4	16.4 ± 2.1	281 ± 25
FA10	a	182 ± 18	165 ± 38	178 ± 29	161.5 ± 8.3	106 ± 10	8.7 ± 2.0	59.1 ± 4.6	67 ± 11	23.5 ± 2.0	294 ± 26
	b	187 ± 18	163 ± 38	177 ± 29	157.6 ± 8.1	110 ± 10	6.8 ± 1.7	60.1 ± 4.7	67 ± 11	23.1 ± 2.0	290 ± 25
FA11	a	132 ± 10	119 ± 10	126.5 ± 5.9	116.5 ± 4.0	132.5 ± 8.4	-	112.9 ± 4.3	122.7 ± 5.6	44.1 ± 2.1	258 ± 14
	b	135 ± 10	128 ± 11	112.1 ± 5.3	107.5 ± 3.9	133.9 ± 8.2	8.8 ± 2.9	107.4 ± 4.4	104.1 ± 4.8	40.4 ± 2.0	235 ± 14
S1	a	149.9 ± 6.5	-	150.9 ± 2.2	-	-	-	45.6 ± 1.5		42.5 ± 2.3	14.2 ± 0.5
	b	163 ± 12	-	143.4 ± 1.6	-	-	-	45.80 ± 0.8		43.2 ± 0.7	15.2 ± 0.3
S2	a	89.2 ± 5.2	96.7 ± 4.8	96.7 ± 4.8	84.9 ± 2.3	-	-	30.0 ± 1.3	30.4 ± 1.4	10.3 ± 0.4	96.1 ± 5.7
S3	a	44 ± 12	34 ± 11	39.1 ± 6.1	37.6 ± 2.8	30 ± 10	2.23 ± 0.61	62.5 ± 4.6	64 ± 10	23.8 ± 3.0	846 ± 73
	b	35.3 ± 7.4	31.7 ± 7.8	32.7 ± 5.0	29.4 ± 1.9	26.5 ± 8.3	-	52.0 ± 3.5	53.6 ± 8.7	19.6 ± 2.4	632 ± 54
S4	a	20.5 ± 5.3	22.6 ± 8.1	16.1 ± 2.6	15.0 ± 1.4	-	-	5.11 ± 0.61	5.7 ± 1.0	14.3 ± 2.9	-
	b	15.3 ± 3.7	23.2 ± 5.0	17.8 ± 2.8	16.6 ± 1.5	-	-	5.53 ± 0.83	5.5 ± 1.0	49.4 ± 6.5	4.0 ± 2.9
S5	a	19.5 ± 4.1	16.1 ± 5.1	16.1 ± 2.5	15.4 ± 1.2	-	-	4.62 ± 0.64	5.01 ± 0.85	4.44 ± 0.77	-
	b	16.4 ± 4.4	19.6 ± 6.6	15.7 ± 2.5	14.2 ± 1.2	-	-	4.22 ± 0.70	4.99 ± 0.84	4.43 ± 0.86	-
SF1	a	-	-	1.7 ± 0.2	-	-	-	-	0.8 ± 0.3	-	99.8 ± 5.2
	b	-	-	1.1 ± 0.4	-	-	-	-	0.8 ± 0.1	-	91.8 ± 4.6
WG1	a	12 ± 1.5	-	10.4 ± 0.3	-	-	-	6.0 ± 0.3		6.72 ± 0.16	1.9 ± 0.1
	b	10.7 ± 1.7	-	7.1 ± 0.3	-	-	-	5.7 ± 0.4		5.84 ± 0.18	1.8 ± 0.1
L1	a	16.1 ± 3.4	-	17.82 ± 0.72	-	-	-	-	-	-	-
	b	18.8 ± 3.5	-	16.13 ± 0.70	-	-	-	-	-	-	-
RM1	a	113 ± 11	100.5 ± 3.8	100.5 ± 3.8	89.2 ± 2.8	-	-	321.3 ± 8.0	287 ± 10	92.1 ± 2.7	55.1 ± 8.5
MK1	a	35.7 ± 7.6	28.3 ± 6.3	30.9 ± 4.8	-	31 ± 10	-	24.1 ± 2.0	23.2 ± 3.8	8.1 ± 1.1	188 ± 17
	b	29.7 ± 6.4	29.0 ± 6.3	25.3 ± 3.9	22.4 ± 1.8	25.8 ± 8.5	-	20.9 ± 1.8	22.8 ± 3.8	6.93 ± 0.92	153 ± 15
MK2	a	34.3 ± 7.5	34.5 ± 7.5	36.0 ± 5.5	30.6 ± 2.0	35 ± 10	-	28.9 ± 2.1	30.2 ± 4.9	8.9 ± 1.1	214 ± 19
	b	33.7 ± 7.4	33.9 ± 7.3	32.8 ± 5.0	29.1 ± 1.9	33 ± 10	-	27.9 ± 2.1	29.9 ± 4.9	8.6 ± 1.1	197 ± 18
PZ1	a	16.6 ± 4.1	14.9 ± 4.7	17.3 ± 3.0	17.1 ± 2.0	18 ± 10	< 2.5	21.4 ± 2.0	22.9 ± 3.9	8.2 ± 1.3	296 ± 28
	b	17.8 ± 4.2	10.9 ± 4.2	15.2 ± 2.4	14.4 ± 1.3	14.5 ± 5.5	< 1.8	19.6 ± 1.6	21.0 ± 3.4	7.5 ± 1.0	267 ± 24
PZ2	a	26.4 ± 6.0	18.7 ± 9.4	20.6 ± 3.4	19.2 ± 2.1	19 ± 14	< 2.3	25.7 ± 2.8	27.4 ± 4.5	9.8 ± 1.4	353 ± 32
	b	24.4 ± 5.4	16.6 ± 5.4	18.1 ± 2.8	17.7 ± 1.5	20.9 ± 7.1	< 1.8	23.7 ± 1.9	24.2 ± 3.9	8.6 ± 1.1	312 ± 28
PZ3	a	23.9 ± 7.6	11.1 ± 6.7	17.0 ± 2.9	16.4 ± 1.9	15.8 ± 8.2	< 2.4	23.9 ± 2.7	25.6 ± 4.3	9.0 ± 1.4	156 ± 16
	b	20.6 ± 4.7	14.9 ± 4.8	19.4 ± 3.0	16.9 ± 1.4	13.4 ± 5.2	< 1.8	24.9 ± 1.9	25.4 ± 4.1	8.8 ± 1.1	156 ± 14
OBBAM1	-	< 3.7	9.1 ± 0.8	9.1 ± 0.8	9.53 ± 0.61	14.5 ± 6.1	-	6.5 ± 1.3	6.0 ± 0.6	1.8 ± 0.1	35898 ± 116
OBFAM1	-	< 10	11.87 ± 0.8	11.87 ± 0.8	10.85 ± 0.64	88 ± 13	-	7.10 ± 1.0	6.3 ± 0.4	2.3 ± 0.3	5852 ± 204

Uncertainties are quoted for a coverage factor k = 2 and are due to measurement uncertainties.

**Table 7:** Activity concentration for the main gamma emitters in the naturally occurring ^238^U, ^235^U, ^232^Th and ^40^K series in different pigments.

Series	^238^U Radiactive Serie					^235^U	^232^Th Radiactive Serie			^40^K
Pigments	^234^Th	^226^Ra	^214^Pb	^214^Bi	^210^Pb		^228^Ac	^212^Pb	^208^Tl	
P1	7.0 ± 0.9	10.9 ± 3.0	12.0 ± 2.0	12.0 ± 2.0	44 ± 13	< 1.6	22.6 ± 1.8	25.4 ± 4.1	10.4 ± 1.4	467 ± 41
P2	<7.4	< 7.7	< 1.8	< 1.9	< 8.1	< 2.3	< 1.7	< 0.7	< 0.5	< 8.8
P3	< 9.0	< 8.1	< 1.6	< 2.1	< 11.4	< 2.5	< 2.6	< 0.9	0.94 ± 0.21	< 7.9
P4	< 6.8	< 5.9	< 1.7	< 1.6	< 9.1	< 1.8	< 1.3	< 0.6	< 0.4	< 4.7
P5	187 ± 39	196 ± 36	232 ± 35	256 ± 15	192 ± 56	< 4.6	13.2 ± 1.5	5.3 ± 1.7	4.07 ± 0.62	301 ± 27
P6	< 11.7	< 14.3	3.7 ± 1.1	< 4.4	< 12.3	< 4.2	< 3.3	< 1.4	< 1.0	< 13.0
P7	< 5.7	< 7.4	1.9 ± 0.5	1.89 ± 0.50	< 6.1	< 1.8	< 1.3	< 0.6	< 0.5	< 5.0

Uncertainties are quoted for a coverage factor k=2 and are due to measurement uncertainties.

**Table 8:** Activity concentration for the main gamma emitters in the naturally occurring ^238^U, ^235^U, ^232^Th and ^40^K series in different aggregates.

Series		^238^U Radiactive Serie					^235^U	^232^Th Radiactive Serie			^40^K
Aggregates		^234^Th	^226^Ra	^214^Pb	^214^Bi	^210^Pb		^228^Ac	^212^Pb	^208^Tl	
A1	a	-	15.4 ± 5.8	4.39 ± 0.82	-	-	-	6.88 ± 0.69	8.1 ± 1.5	3.00 ± 0.30	173 ± 17
	b	-	-	4.96 ± 0.90	-	7.2 ± 1.5	-	7.14 ± 0.69	7.7 ± 1.3	2.77 ± 0.28	141 ± 12
A2	a	208 ± 20	213 ± 36	213 ± 34	193 ± 10	187 ± 16	10.6 ± 1.6	71.2 ± 5.4	79 ± 13	27.1 ± 2.3	1149 ± 98
A3	a	155 ± 20	153 ± 38	150 ± 24	138.5 ± 7.7	152 ± 18	-	105.4 ± 8.8	114 ± 19	39.9 ± 3.6	1090 ± 94
A4	a	133 ± 16	131 ± 31	128 ± 21	118.4 ± 6.3	118 ± 15	6.9 ± 1.7	172 ± 13	183 ± 29	63.4 ± 5.3	1195 ± 102
A5	a	37.5 ± 9.0	24.7 ± 9.3	27.6 ± 4.2	26.0 ± 1.9	25.9 ± 7.7	-	48.3 ± 3.4	50.9 ± 8.2	18.1 ± 2.2	560 ± 48
	b	30.0 ± 6.3	24.9 ± 6.3	27.1 ± 4.1	± 23.6 ± 1.5	26.7 ± 8.2	-	45.7 ± 3.0	48.3 ± 7.8	17.3 ± 2.1	518 ± 44
A6	a	140 ± 31	125 ± 34	135 ± 21	127.2 ± 8.0	15.6 ± 5.8	-	34.6 ± 3.5	37.8 ± 6.3	13.4 ± 1.9	76 ± 10
	b	139 ± 29	131 ± 30	139 ± 21	121.8 ± 7.2	18.6 ± 7.3	8.0 ± 2.3	34.5 ± 2.6	38.8 ± 6.3	13.5 ± 1.7	81.7 ± 8.5

Uncertainties are quoted for a coverage factor k=2 and are due to measurement uncertainties.

#### 1.2.1 Cements

Activity concentrations of ^238^U, ^235^U and ^232^Th series, and ^40^K in commercial cements are shown in Table 5.

#### 1.2.2 Supplementary Cementitious Materials (SCM)

Results obtained from gamma spectroscopy in ^238^U, ^235^U and ^232^Th series and ^40^K series for 37 *Supplementary Cementitious Materials (SCM)* are presented in Table 6.

#### 1.2.3 Pigments used in mortar and concrete technology

Activity concentrations of ^238^U, ^235^U and ^232^Th series and ^40^K in seven different pigments are shown in Table 7.

#### 1.2.4 Aggregates used in mortar and concretes preparation

Activity concentrations of ^238^U, ^235^U and ^232^Th series and ^40^K for aggregates are shown in Table 8.

## 2 Materials and Methods

### 2.1 Materials

In this work, a total of 56 samples of components used in cement-based materials have been studied.

Commercial Cements: 16 cements with different chemical and mineralogical composition.

a Six Type I cements (CEM I 42.5R y CEM I 52.5R) from different cement factories (samples C1-C6). according to European Standard EN-197-1-2011 [3]
b Three blended cements: One CEM II/A-L with a maximum of 20% of limestone (sample C7), one CEM II/B-V with coal fly ash (FA) content around 21–35% (sample C8) and one CEM III/B with high content (around 66–80%) of blast furnace slag (sample C9). All of them according to European Standard EN-197-1-2011 [3].
c One CEM I 52,5 S/R, sulphate-resistant, which therefore has a lower proportion of tricalcium aluminate (C_3_A) (sample C10) [3]
d Two white cements (samples C11 and C12) with a very low Fe_2_O_3_ content and without TiO_2_. as per standard UNE 80305:2012 [4]
e Three calcium aluminate cements (CAC) from different factories, with very different chemical and mineralogical composition compared to a Portland cement, whose main component is monocalcium aluminate (CA) (samples C13-C15). This type of cement complies with European standard EN 14647-2006 [5]
f One calcium sulfoaluminate cement with a very different chemical composition and with a very low SiO_2_ content while higher sulphate proportion.

Supplementary Cementitious Materials: 27 SCMs from very different sources, some of them commonly used in the preparation of cement-based materials.

a Fly ash (FA): Eleven coal fly ashes from seven different thermoelectric power plants, with different composition and proportion of unburned material (samples FA1-FA11)
b Slags: Three vitreous blast furnace slags with different origins (samples S1-S3) and two steel slags (samples S4 and S5). Slags are by-products of iron and steel-making production.
c One silica fume (sample SF1) which is a by-product originated in the reduction of quartz with coal, in electric arc furnaces for the production of silicon and ferrosilicon.
d One waste glass (sample WG1) from urban glass recycling waste.
e One limestone (sample L1)
f One red mud (sample RM1) that is a solid waste with high iron content, generated in the industrial process to produce alumina from bauxite.
g Two samples of metacaolin (sample MK1 and MK2) resulting from the calcination of kaolinitic clays.
h Three pozzolans, with different origin and chemical composition (samples PZ1-PZ3)
i Two olive biomass samples prepared from the mixture of various biomass bottom ashes (sample OBBAM1) or biomass fly ashes (OBFAM1).

Pigments: Seven pigments (samples P1-P7) commonly used in construction, with different colour and chemical composition. One of them (P6) has a high organic matter content.

Aggregates: Six aggregates with maximum particle size of 2 mm.

a One standardized siliceous aggregate according to EN 196-1 (sample A1).
b Three granitic aggregates of different geographical origin (samples A2-A4).
c Two volcanic aggregates (samples A5 and A6).

### 2.2 Methods

#### 2.2.1 Chemical characterization

The chemical characterization of all the materials was determined on a S8 Tiger Bruker X-ray fluorescence (XRF) spectrometer. Loss on ignition calculated as per UNE-EN196-2:2014 [1], was also determined. All samples were dried in an oven at 105 °C for 24 h and then, they were milled to ensure a particle size below 63 microns. The oxide composition of the samples, determined by XRF, was carried out on pressed pellets. For this, 2 g of sample were used with 0.2 g of Hoechst wax C on a boric acid support, in an aluminum capsule. For the preparation of fuse beads, 1 g of sample was mixed with 10 g of melting agent (66% Lithium Tetraborate/34% Lithium Metaborate) and LiBr stripper.

#### 2.2.2 Gamma spectroscopy analysis

Radiological measurements of the samples were performed by gamma spectrometry with a counting time of 80,000 s, using three High-purity Germanium detectors (one of them is coaxial Type p, and the others are BEGe Type -Broad Energy Germanium detectors).

The detectors have an active surface from 26 to 38 cm 2 and 2 keV resolution for ^60^Co and intrinsic efficiencies from 30% to 100%. They are protected with 15 cm iron shield. Main characteristics of both types of Ge detectors are shown in Table 9.

**Table 9:** Characteristics of GXGe and BEGe high purity detectors used in gamma measurements.

Parameter	Detector 07	Detector 90
Model	GX10022	BE50360
Type	Extended Range Coaxial	Broad Energy
Resolution at 1.33 MeV (KeV)	2.04	1.84
Relative efficiency at 1.33 MeV (KeV)	115.7	48.0
Crystal diameter (mm)	84	80
Crystal length (mm)	72	30
Outer shielding (cm)	15	15
Inner shielding 1^st^ layer (Cu) (mm)	3.0	2.5
Inner shielding 2ndf layer (Zn) (mm)	-	1.5
Inner space shielding (dm^2^)	25	25
Shielding composition	Fe	Pb

The gamma detectors are connected to two electronic chains consisting of: two amplifiers, two Analog-to-Digital Converters (ADCs), 2 High Voltage Power Supplies and 2 AIM modules all from Canberra Industries. The AIMs modules have the function of communicating the electronic chain with the PC allowing it to: (1) control the parameters of the detector and electronic chain and (2) acquire the spectra. Once the spectra were acquired they were analysed with Genie 2000 software [6] and a MS Excel spreadsheet [7]. The efficiency as a function of energy was computed by using the mathematical code based on Monte Carlo LabSOCs. The parameters required for this calibration were (1) dimensions and composition of the detector components, (2) dimensions of the sample container and (3) chemical composition and density of the sample matrix [8].

All samples were measured in specific polypropylene sample container (cylinders 75.4 mm of diameter, 31 mm height) sealed to avoid ^222^Rn loses. Each container was totally filled with the sample and the measurements were carried out after 21 days to ensure secular equilibrium between radionuclides and their progeny [9], [10], [11], [12]. The samples were measured in duplicate, except in cases where a sufficient quantity of sample was not available.

Radionuclides occurring in natural decay chains headed by ^238^U (^226^Ra), ^232^Th and ^40^K were determined in the samples.

^238^U serie measurements were based on the detection of emissions of their daughter nuclides, ^234^Th (63 keV), ^226^Ra (186.1 keV), ^214^Pb (351 keV) and ^214^Bi (609, 1120 and 1765 keV).

^232^Th determination was determined on emissions of their daughter nuclides, ^228^Ac (911 and 969 keV), ^212^Pb (238 keV) and ^208^Tl (583 keV) respectively, since their own gamma emitting lines are not of sufficient intense (^232^Th at 63.8 keV) or present significant interferences with other naturally occurring radionuclides.

^235^U (144 and 163 KeV) and ^40^K (1460.8 keV) were directly measured by their emissions lines.

## Declaration of Competing Interest

The authors declare that they have no known competing financial interests or personal relationships which have, or could be perceived to have, influenced the work reported in this article.
